# A Case of Roof‐Dependent Atrial Flutter With an Endocardial Narrow Channel Successfully Visualized by the Peak Frequency Analysis

**DOI:** 10.1002/joa3.70294

**Published:** 2026-02-16

**Authors:** Tokuma Kawabata, Yoshimori An, Kazuhiro Ogura, Shiho Enomoto, Toshiaki Izumi

**Affiliations:** ^1^ Department of Cardiology Osaka Saiseikai Noe Hospital Osaka Osaka Japan

**Keywords:** near‐ and far‐field electrogram, omnipolar technology, peak frequency mapping, roof line ablation, roof‐dependent atrial flutter

## Abstract

Roof‐dependent atrial flutter (AFL) can sometimes be challenging to treat with endocardial catheter ablation due to the thickness of the atrial myocardium and the separation of outer epicardial conductive fibers from the endocardial myocardium. This case highlights the utility of peak frequency (PF) analysis during the AFL, which can visualize a narrow endocardial channel and help physicians select an optimal therapeutic target to achieve a transmural line of block.

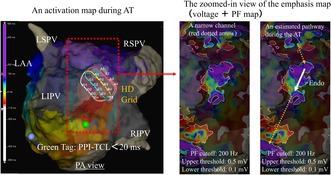

A 72‐year‐old woman with a chief complaint of palpitations was admitted to our hospital with atrial tachycardia (AT). She had previously undergone radiofrequency catheter ablation for atrial fibrillation (AF) about 10 years ago at another institution. At the beginning of the second ablation procedure, an AT with a total cycle length (TCL) of 256 ms was sustained, and electrode catheters were placed (Figure 1A). A voltage map during the AT was created using the Advisor HD‐Grid (HDG) Mapping Catheter Sensor Enabled (Abbott) and the TactiCath Contact Force Ablation Catheter, Sensor Enabled in the 3D mapping of the EnSite X EP System (Abbott, Abbott Park, IL, USA). It showed durable lesions of pulmonary vein isolation (PVI) created in the previous 1^st^ session and low‐voltage (< 0.5 mV) zones on the roof and bottom side of the LA posterior wall (Figure 1B). We diagnosed the AT as an LA roof‐dependent atrial flutter (AFL) based on the findings of the activation map during AT (Figure 1C) and the post‐pacing interval (PPI) (Figure 1E). The emphasis map (a combination of voltage and peak frequency [PF] maps with a cutoff of 200 Hz) showed a narrow channel of the AT circuit with higher PF values in the LA posterior wall, which was considered a breakthrough portion on the endocardial side (Figure 2A and 2B). The AT was terminated by delivering radiofrequency energy (power of 30 W and contact force of 10–20 g, targeting a lesion size index of 4.5) at two points covering this narrow channel (Figure 2C). After tachycardia was terminated, we observed the low‐voltage areas of local unipolar and bipolar potential adjacent to the two ablated points, suggesting that the block line may have been completed. Then, we confirmed the bidirectional block by creating activation mapping, using the HDG catheter, while pacing with a high output (10 V and 1.0 ms) from the ablation catheter positioned on the opposite side across it. Finally, programmed electrical stimulation with isoproterenol infusion did not induce atrial arrhythmia. During a 12‐month follow‐up, the patient remained free from recurrent atrial tachyarrhythmia.

**FIGURE 1:**
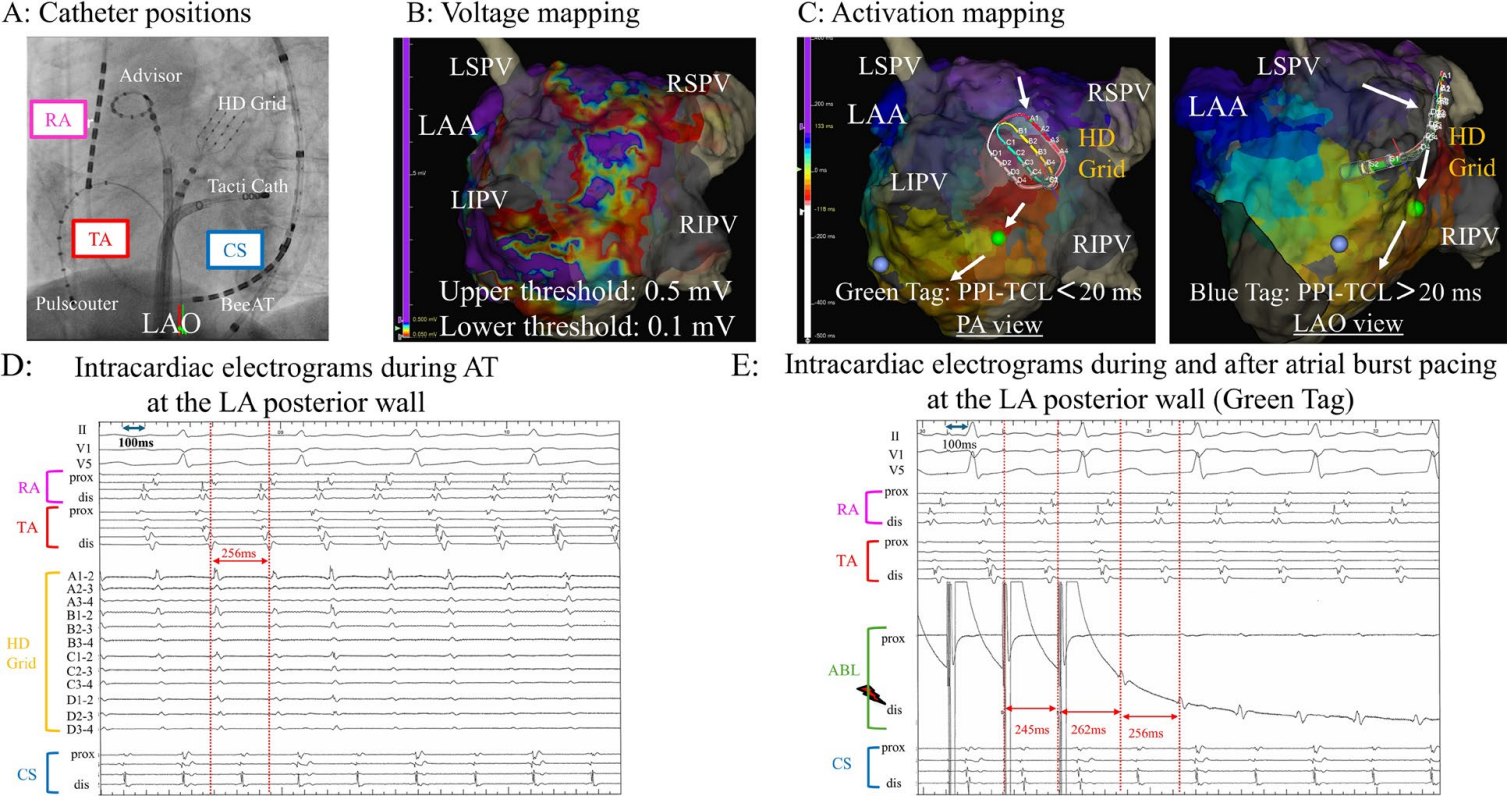
(A) Catheter positions for the in situ recording of AT in fluoroscopic images. (B) Three‐dimensional voltage map of the LA acquired using the EnSite X system during the AT. A bipolar electrogram amplitude of < 0.5 mV was defined as low voltage, and sites with low voltage < 0.1 mV were designated as scarred areas. (C) A three‐dimensional activation map of the LA acquired using the EnSite X system during AT showing that it propagates from the upper to the lower part of the LA posterior wall. (D) Intracardiac electrogram recorded during AT with a cycle length (CL) of 256 ms. (E) Intracardiac electrogram recorded during pacing with a pacing CL of 245 ms at the distal tip of the ablation catheter (ABL), positioned in the LA posterior wall (indicated by the green tag in Figure 1B) during AT, entrained the tachycardia with concealed fusion and a post‐pacing interval of 262 ms (AT‐TCL + 6 ms). AT: Atrial tachycardia; LA: Left atrium; RA: Right atrium; CS: Coronary sinus; TA: Tricuspid annulus; LAO: Left anterior oblique; LSPV: Left superior pulmonary vein; LIPV: Left inferior pulmonary vein; RSPV: Right superior pulmonary vein; RIPV: Right inferior pulmonary vein; LAA: Left atrial appendage; PA: Posterior anterior; PPI: Post‐pacing interval; TCL: Total cycle length.

**FIGURE 2:**
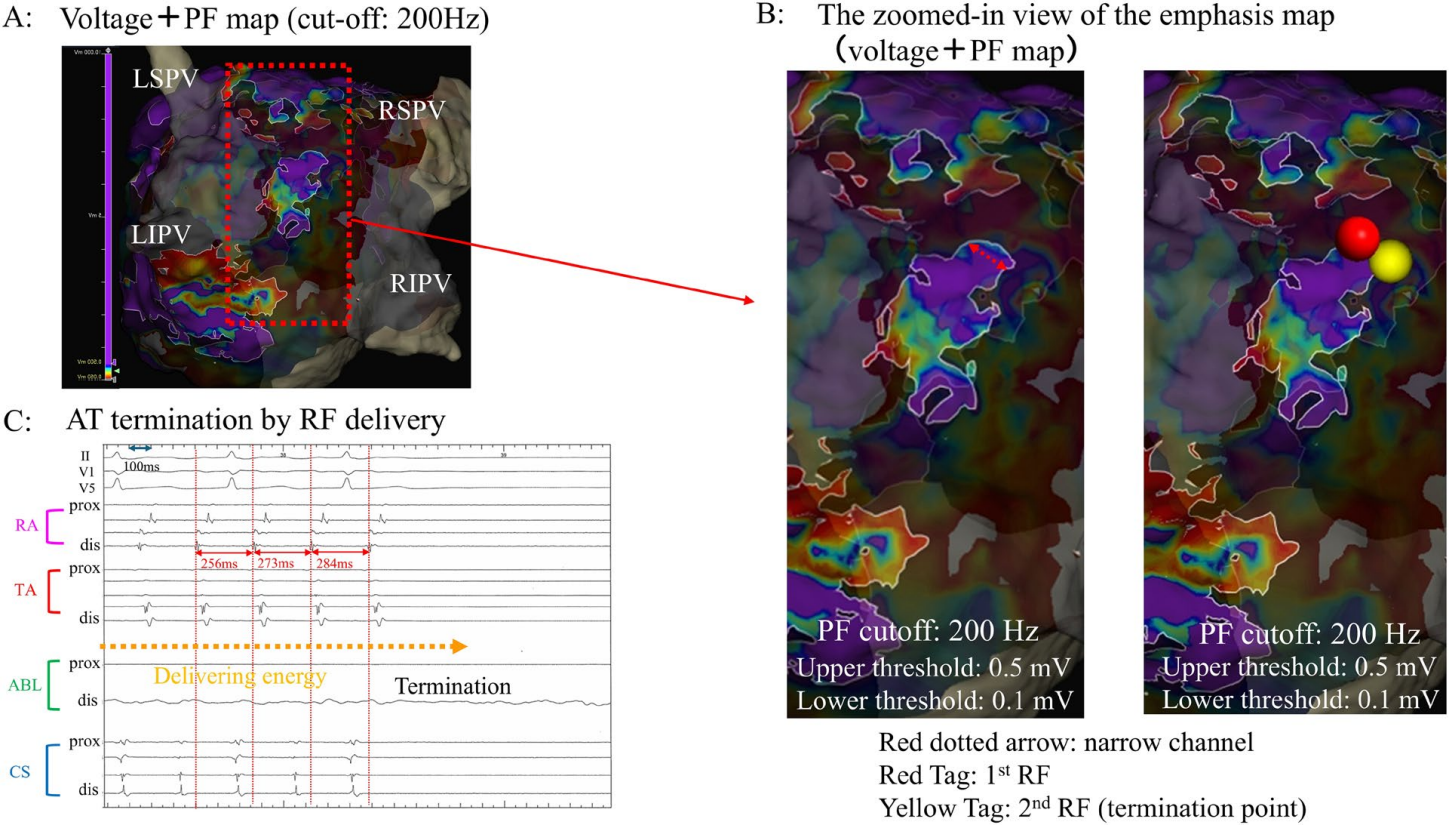
(A) Three‐dimensional emphasis map combining voltage and peak frequency mapping (cut‐off: 200 Hz) during AT. (B) Emphasis map showing a narrow channel (indicated by the red dotted arrow) with high‐frequency sites in the LA posterior wall on the AT circuit, which was considered a breakthrough portion from the far‐field (subendocardial or epicardial) to the near‐field endocardial side. (C) AT was terminated by delivering radiofrequency energy at two points (red tag and termination yellow tag) covering this narrow channel by slowing the AT cycle length from 256 ms to 273 ms, and then to 284 ms. PF = peak frequency; RF = radiofrequency. Other abbreviations are as in Figure 1.

Roof‐dependent AFL is a major reentrant tachyarrhythmia that propagates in the LA [1]. Typically, LA roof line ablation is performed to block this macro‐reentrant tachycardia. However, it is sometimes difficult to create a transmural block of the roof line from the endocardial side. This is partly due to the involvement of epicardial conduction, such as the septo‐pulmonary bundle (SPB). The anatomy and course of the SPB are associated with the failure to block the roof line, possibly because the layers of interposed adipose tissue separated from the atrial endocardium may protect the SPB from thermal ablation energy [2].

Ensite OT Near Field algorithm can automatically annotate the highest PF associated with the local electrogram, resulting in accurate near‐field potential annotation. A high PF value may reflect the existence of live endocardial tissues and the proximity of the electrodes. By contrast, a low PF value may reflect local electrograms of the subendocardial or epicardial layers [3]. Recently, Takamiya et al. reported that the PF is significantly larger in the superior vena cava and PVs, where muscle volume is scarce, compared to the atrial body with multilayered myofibers [4]. These findings suggest that a significant muscle mass further from the endocardial surface could increase far‐field effects and consequently decrease PF. The higher PF value may indicate myocardial thinness and closeness to the endocardial surface of the tachycardia circuit (Figure 3). Yamagami et al. reported the efficacy of the PF value of the local potentials mapped with the HDG catheter during the LA appendage pacing for creation of the LA roofline [5]. As with their data while pacing from the LA appendage, our present case showed that the PF value of the local potential during the AT could also provide helpful information on the endocardial narrow channel of the AT circuit in 3D activation mappings. Delivering radiofrequency energy to high‐frequency zones through the LA posterior wall can be a useful strategy for terminating the tachycardia and minimizing the number of ablation applications.

**FIGURE 3:**
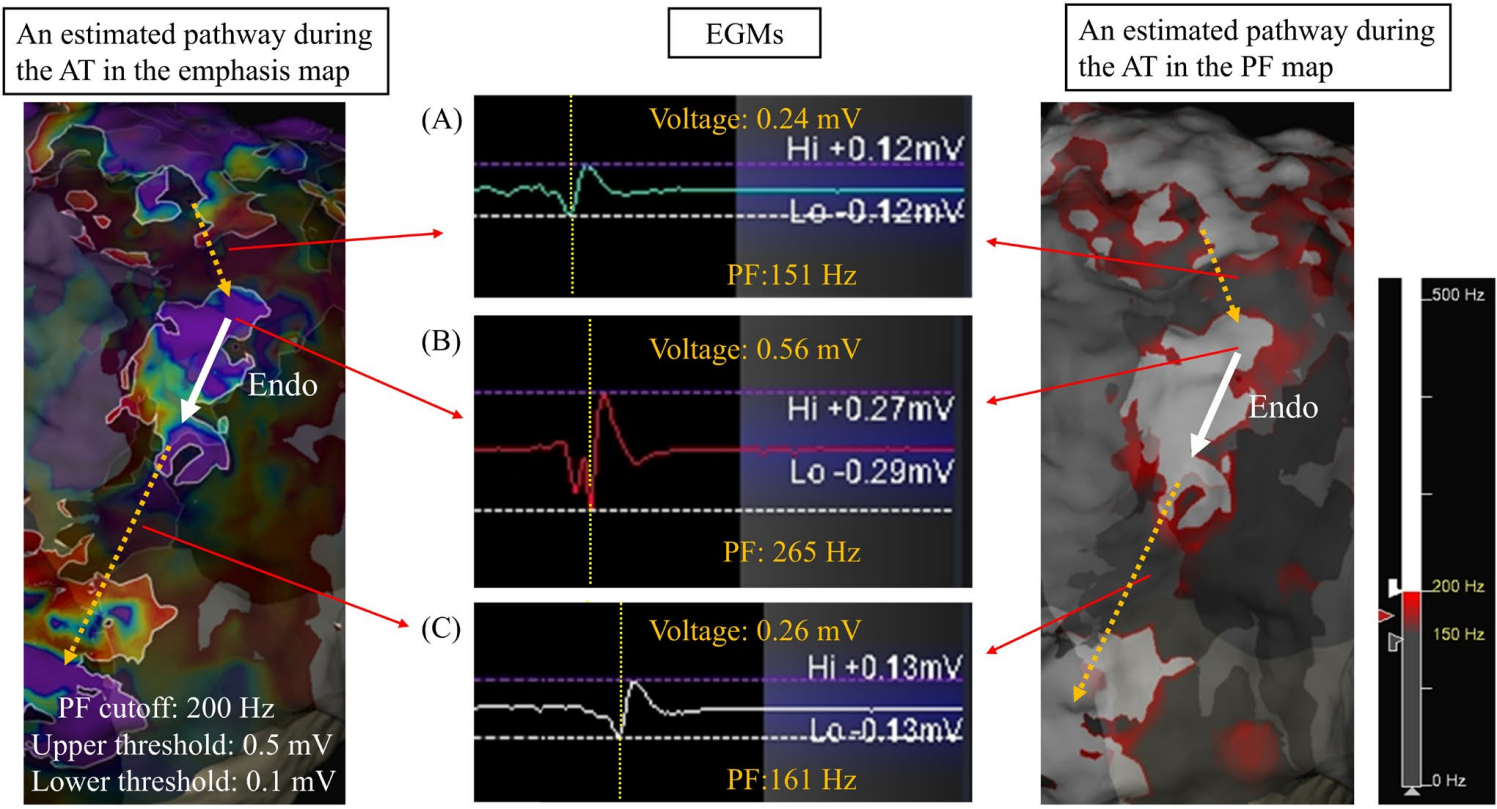
The PF value at the termination point (265 Hz, as shown in B) was higher than that at other local electrograms (as shown in A and C) in the LA posterior wall. The AT circuit considered from the PF value at the LA posterior wall was that propagation from LA roof to a narrow channel by the subendocardial or epicardial conduction (orange dotted line), the endocardial propagation between the high frequency area (white solid line), and again, the subendocardial or epicardial propagation to the LA bottom (orange dotted line) (shown by left panel). EGM = electrogram. Other abbreviations are as in Figures 1 and 2.

There were some limitations to the electrophysiological study in this case. First, we could not assess LA myocardial thickness using CT or MRI. The thickness of the myocardium at the successful termination point is unclear. Second, the optimal PF cutoff value to distinguish between epicardial and endocardial local potentials in the LA posterior wall remains unknown because of significant individual variability in the PF. For this reason, in our present case, the cutoff PF value was adjusted to 200 Hz by decreasing in 10‐Hz increments from a nominal frequency of 250 Hz to visualize a narrow endocardial channel of the AT circuit. The relationship between myocardial thickness and PF values in the LA posterior wall requires further investigation. However, we believe that we can optimize the first target site to create a transmural line of block during the AT by adjusting the PF cutoff value to identify the mapped points with higher PF values compared to the surroundings. Finally, the frequency and voltage of the local electrograms may be affected by the contact force of the catheter electrodes touching the myocardial tissue. It may sometimes be challenging to accurately assess the electrode‐tissue contact when using the HDG catheter, although we ensured the endocardial surface of the LA using an ablation catheter with a contact force sensor.

In conclusion, we encountered a case of roof‐dependent AFL that could be terminated by delivering radiofrequency energy to a narrow channel visualized by PF mapping. In tachycardia mediated by epicardial conduction, it may be useful to visualize regions with high PF values, which indicate myocardial thinness and proximity to the endocardial surface of the tachycardia circuit.

## Funding

The authors have nothing to report.

## Consent

Patient consent for publication was obtained.

## Conflicts of Interest

The authors declare no conflicts of interest.

## Data Availability

The data that support the findings of this study are available from the corresponding author upon reasonable request.
